# Climate and biocrust types jointly regulate soil multifunctionality and quality in drylands: evidence from the Gurbantunggut Desert

**DOI:** 10.3389/fpls.2026.1670208

**Published:** 2026-02-10

**Authors:** Yonggang Li, Yingjie Gao, Yunjie Huang, Yongxing Lu, Benfeng Yin, Xiaobing Zhou, Hao Yu, Yuanming Zhang

**Affiliations:** 1 School of Plant Protection and Environment, Henan Institute of Science and Technology, Xinxiang, China; 2 Key Laboratory of Ecological Safety and Sustainable Development in Arid Lands, Xinjiang Institute of Ecology and Geography, Chinese Academy of Sciences, Urumqi, China

**Keywords:** biocrusts, desert, soil multifunctionality, soil quality index, spatial variability of soil multifunctionality

## Abstract

Soil multifunctionality (SMF) and the soil quality index (SQI) are essential indicators of soil function, productivity, and health. Additionally, the spatial variability of soil multifunctionality (SVM) signifies soil heterogeneity. Biological soil crusts (Biocrusts) can affect these indicators. However, there is little information about the role of biocrusts in regulating the response of multiple ecosystem functions to climate change. We evaluated the relative importance of climate, soil environment, and biocrusts variables as drivers of SMF, SQI, and SVM at 74 sites in the Gurbantunggut Desert. Soil SMF, and SQI increase with the coverage of lichen and moss crust. Biocrusts index, SMF and SQI increase with an increase in the mean annual temperature. Biocrusts index, SMF and SQI increase first with an increase in mean annual precipitation (MAP)< 163 mm and then decrease. SVM display a significant decreasing trend with the increase of MAP. The structural equation model (SEM) demonstrate that the spatial distribution can significantly influence the biocrusts, soil SQI and SVM. Biocrusts has a significant positive influence on soil SMF (0.47)and SQI (0.31). Soil SMF has a significant negative effect on SVM (-0.50), and SQI (0.59) has a significant positive effect. We provide the first quantitative evidence that biocrust type and a 163 mm precipitation threshold govern SMF through opposing direct vs. indirect temperature pathways, offering a predictive rule-of-thumb for dryland management under climate change. The findings contribute decidedly to our understanding of the patterns and mechanisms driving SMF, SQI, and SVM in drylands, which is important for predicting changes in ecosystem function under climate change.

## 1 Introduction

When considering terrestrial ecosystems, most ecosystem functions are provided by soils. Soils (abiotic and biotic components) directly contribute to several ecosystem functions such as nutrient and water cycling and biodiversity. Therefore, soil carries immense biodiversity and support key ecosystem processes essential for life (Eldridge et al., 2020; Guerra et al., 2020). These processes encompass nutrient cycling (Hu et al., 2023; Ochoa-Hueso et al., 2018), trophic interactions (Hagh-Doust et al., 2023), individual plant performance, and competitive ability (Day et al., 2003; Huang et al., 2023), as well as community-level productivity (Garcia-Callejas et al., 2023). Soils can be characterized by different physical, chemical and biological properties (Bouma, 2002). These soil properties influence soil multifunctionality (SMF), soil quality index (SQI), and, subsequently, ecosystem processes.

SQI is a comprehensive metric used to evaluate the health status of soil and its capacity to support specific functions (e.g., agricultural production, environmental protection). It quantifies soil quality by integrating multiple physical, chemical, and biological indicators of soil. SQI can assess the capacity of the soil to maintain and support its productivity and health in the relevant ecosystem by combining several physical, chemical, and biological properties (Lal et al., 2021). The SQI assessment would have important implications for resource management and provides a quantitative method for evaluating the soil quality at the soil type-scale (Kafei et al., 2023; Khosravi et al., 2023). Changes in SQI are linked to interactions among ecosystem processes, potentially disrupting the equilibrium between soil physiochemical, microbial, and biochemical processes (Hedo de Santiago et al., 2016). Studies indicate that external materials, like fertilizers, can modify microbial metabolic traits by changing soil nutrient levels, subsequently influencing soil SMF (Huo et al., 2024; Yang et al., 2023). Therefore, the SMF and SQI are calculated from soil properties that respond quickly to grazing, cultivation, or restoration, any change in land use is immediately reflected in the index values (Zornoza et al., 2015). Using multivariate indicators is the best way to estimate SQI because they provide a broader view of the situation than individual property analyses.

In terrestrial ecosystems, there exists a widespread pattern of uneven soil characteristic distribution, which plays a pivotal role in regulating diverse ecosystem processes. Notably, this pattern is particularly pronounced in dryland ecosystems, will directly affect the spatial heterogeneity of desert soil. The spatial variation in soil multifunctionality (SVM) plays a key role in the sustainability and stability of ecosystem functions (Juhos et al., 2016). SVM refers to the non-uniform distribution of SMF (i.e., the ability of soil to simultaneously support multiple ecosystem functions) across space. This variation can occur at different scales. The SVM in drylands is likely to be caused by the significant multifunctional differences between vegetated patches, in which plants largely drive biological processes such as litter decomposition, nutrient cycling. In unvegetated areas physical processes such as wind and water play a larger role in SMF than biotic processes (Durán et al., 2018; Li et al., 2007; Zheng et al., 2019). The SVM is influenced by the complex interplay of biotic and abiotic processes (Allington and Valone, 2014; Ochoa-Hueso et al., 2018). Furthermore, climate changes can also affect the variability in soil functions among different vegetation patch types by altering patch patterns (Durán et al., 2018). Due to the sensitivity of drylands to climate change, increasing aridity and temperature would lead to change in vegetation and soil properties that could adversely affect SVM in these areas worldwide. Therefore, climate change and plant cover are associated with alterations of the SVM in drylands (Ding and Eldridge, 2021; Durán et al., 2018). According to recent studies, increasing aridity is correlated with decreasing plant cover and richness, and increasing woody vegetation encroachment rates across the globe (Delgado-Baquerizo et al., 2013; Vicente-Serrano et al., 2012), which would likely lead to an increase in SVM. Evaluating the effect of environmental factors on desert soil SQI and SVM is crucial not only for understanding the processes of desert ecosystems but also for developing practical and sensitive multivariate indices that can be used as management tools in desert regions.

Drylands are crucial for global sustainability, as they constitute 45% of the earth’s land surface (Reynolds et al., 2007), where many vascular plants are restricted due to the shortage of precipitation; however, biocrusts are widespread. Recent estimates indicate that biocrusts currently cover approximately 30% of dryland soils, constituting around 12% of the earth’s terrestrial surface (Rodriguez-Caballero et al., 2018). Biocrusts are composed of cyanobacteria, green algae, lichens, mosses, and other organisms related to soil particles that play essential fundamental roles in arid and semiarid regions (Xiao et al., 2022), including carbon and nitrogen cycling (Bowker et al., 2013; Li et al., 2019), surface energy balance (Couradeau et al., 2016; Rodriguez-Caballero et al., 2018; Rutherford et al., 2017), erosion (Cantón et al., 2014; Chamizo et al., 2017) and water redistribution (Bowker et al., 2013; Kidron and Büdel, 2014), affecting the colonization and development of vascular plants (Langhans et al., 2009; Li et al., 2005), and supplying habitats for other microorganisms and protozoa (Liu et al., 2017).

The biocrusts types can significantly influence the characteristics of soil nutrient cycling (Gao et al., 2018). There is a distinct difference between soil physicochemical properties, microbial activities, and community compositions of different types of biocrusts (Niu et al., 2017; Xu et al., 2021, Xu et al., 2020; Zhang et al., 2018, Zhang et al., 2016). In different types of biocrusts, soil fungal and bacterial communities have different responses and undertake important roles in maintaining nutrient cycling and SMF (Xu et al., 2021, Xu et al., 2020). There are significant differences in the physical and chemical characteristics of the underlying soil under different types of biocrusts, which will influence soil microbial diversity and activity (Lan et al., 2012; Li et al., 2020). Biocrusts notably enhance the content of C, N, and P in surface soil, and change soil nutrient stoichiometry (Baumann et al., 2021). Cyanobacteria vary significantly in diversity, biomass, and species composition due to resource accumulation among different biocrusts types (Lan et al., 2012; Wu et al., 2009). The moss crusts show higher photosynthesis and N_2_ fixation activities than the cyanobacterial and lichen crusts (Housman et al., 2006; Lan et al., 2012). Similarly, nitrogenase activity is higher in cyanobacterial and lichen crusts than in moss crusts (Pushkareva et al., 2017; Wu et al., 2009). Biocrust development exerted direct effects on SMF in arid regions, but only indirect effects through changing soil microbial biomass carbon in semi-arid regions (Su et al., 2021). Moss crusts significantly influence soil SMF. For instance, moss crusts alter phosphorus functionality and enhance phosphorus cycling in the ecosystem (Li et al., 2024). Therefore, biocrusts actively participate in soil surface heterogeneity dynamics in terms of biological diversity, soil function and physicochemical properties associated with their spatial structure (Ettema and Wardle, 2002).

Biocrusts are generally located within the uppermost millimeters of the soil surface (Weber et al., 2022) where they are more likely to be affected by environmental factors, such as soil water, nutrients, temperature, and radiation intensity (Grote et al., 2010; Navas Romero et al., 2020). As a result, the soil nutrients in the soil under biocrusts patches are also affected by environmental changes and are considerably higher than those in soil without biocrusts (Li et al., 2019; Tao et al., 2020). Some studies have explored the effects of biocrusts type on soil SMF (Li et al., 2019, Li et al., 2024; Su et al., 2021). However, it is not clear whether the SVM and SQI in desert soil are affected by climate and biocrusts type in the temperate desert. Additionally, does climate affect the SVM, SQI and SMF of desert soils by influencing biocrusts type. To evaluate the role and relative importance of climate and biocrusts type on the soil SMF, SQI, and SVM in desert ecosystems, we collected 74 sites from the Gurbantunggut Desert. Furthermore, previous studies also found that the climate can significantly influence the growth and distribution of different biocrusts types, as well as the physical and chemical properties and functions of soil (Li et al., 2023, Li et al., 2019). The biomass of moss crusts is 3 to 5 times greater than that of algae-lichen crusts (Zhang et al., 2022). Additionally, their thickness and nifH nitrogenase activity are 2 times and 2.3 times higher, respectively. The pseudoroots and polysaccharide carbon input from moss crusts significantly enhance surface SOC and available nitrogen, directly increasing the importance of “nutrient cycling” in SMF. Thus, we hypothesized that 1) Moss crusts increase SMF and SQI more than algal-lichen crusts, with the largest gains in nutrient-cycling functions; 2) Climate can directly influence soil SVM, SMF, and SQI, and can also indirectly affect SMF, SVM, and SQI by altering the type of biocrusts.

## 2 Materials and methods

### 2.1 Study area

This study was conducted in the Gurbantunggut Desert (44°11′-46°20′ N, 84°31′-90°00′ E, 300–600 m a.s.l.), which is situated in the center of the Jungger Basin, Central Asia. With a total area of 4.88 × 10^4^ km^2^, it is the largest fixed and semi-fixed desert in China. Moist air currents from the Indian Ocean are blocked by the Himalayas and fail to reach this area, causing a vast expanse of arid terrain. The mean annual precipitation (MAP) in the desert is around 103–229 mm (**Supplementary Figure S2**) with gradient distribution (Zhang et al., 2007). The mean annual temperature (MAT) is 6-8°C, while the potential mean annual evaporation is estimated at 2606.6 mm (Zhang et al., 2007). The mean annual wind speed (MAW) of 2–4 m/s. With a mean wind speed of 11.17 m/s, late spring is the windiest time of year. There is prevailing wind from the northwest, northwest and north direction. Soil moisture is about 0.5-2% and the temperature is about 50-60°C. According to nature-reserve management records (1994–2022), zero-grazing policy, and remote-sensing evidence of negligible livestock traces, the study area has experienced no significant anthropogenic disturbance for at least the past three decades. The soil type is an Arenic soil and subclasses of Medium sand (Li et al., 2024).

The distribution of different types of biocrusts was found to be uneven in this desert, with no observed seasonal differences. On the dunes, the species composition varied in different biocrusts types, which could be classified into three groups: algal, lichen, and moss crusts (**Supplementary Figure S2**). Algal crusts are weakly consolidated, soft, and readily decomposed; they often include fungal or cyanobacterial hyphae and are composed mainly of fungi and/or cyanobacteria. Lichens consist of a symbiotic association between heterotrophic fungi and autotrophic partners such as cyanobacteria or green algae. Moss crusts are characterized by short, hairlike extensions and appear brown when dry and green to brown−green when moist. The type and coverage of biocrusts also vary in different regions with climate change. In this study, algal crust and lichen crust generally coexist in biocrusts, so we consider them as one soil type. Previous studies have shown that algae and lichen crusts have no significant differences in key functional indicators such as soil carbon and nitrogen sequestration, surface aggregate stability, and enzyme activity, and their functional contributions are significantly lower than moss crusts; Recently, large-scale biological crust research in arid regions has generally adopted the “algae lichen crust” merging treatment to avoid classification uncertainty caused by visual discrimination errors. Therefore, we selected two biocrusts types: algal, lichen mixed crust and moss crust in our study (**Supplementary Figures S2**–**S4**).

### 2.2 Sample collection and processing

**Figure 1:**
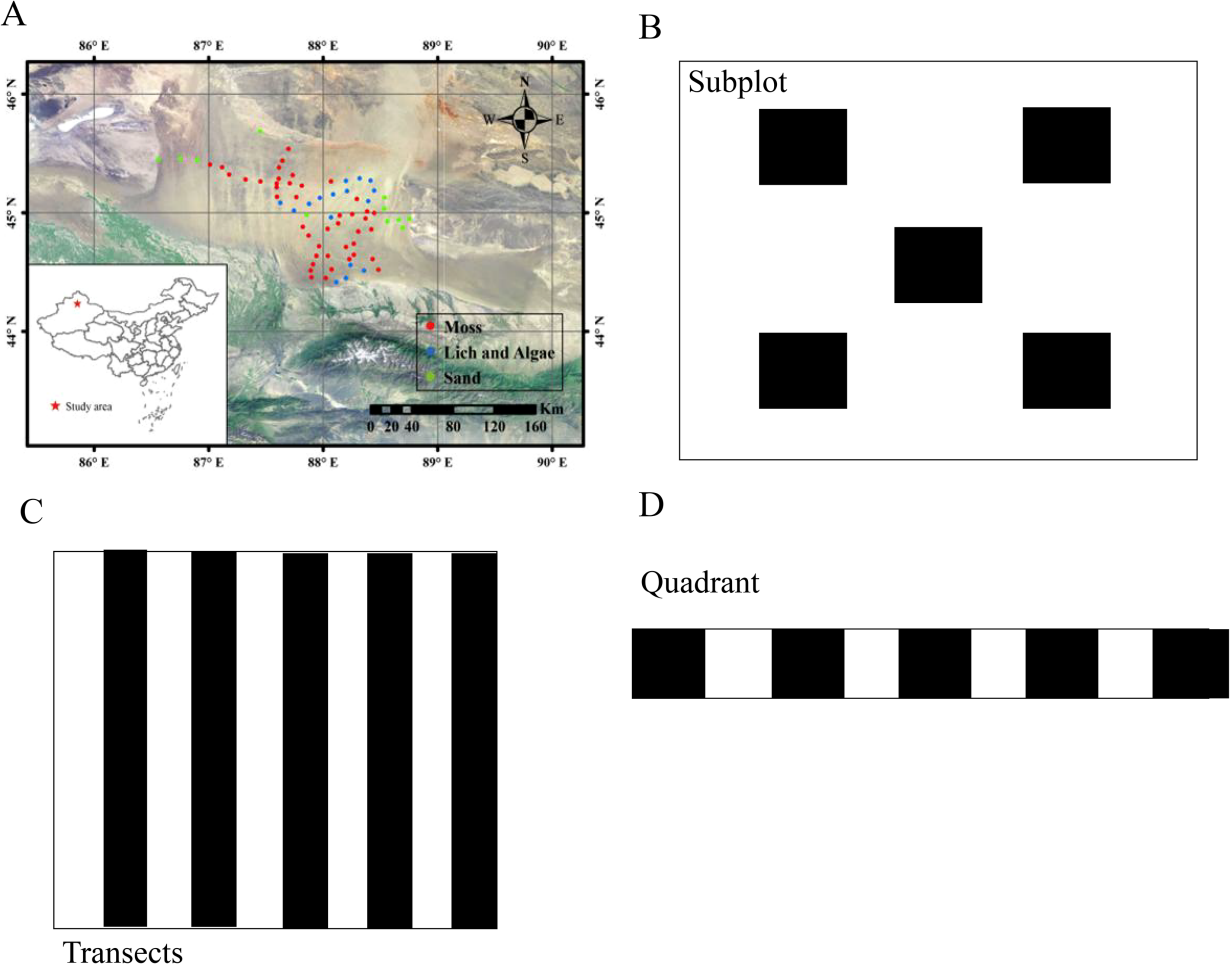
Site distributed of sample collection in Gurbantünggüt Desert, Northwest China. **(A)** displays a specific study area in China, marked with red, green, and blue dots indicating moss, lichens and algae, and sand. **(B)** details a subplot arrangement with five black squares. **(C)** shows transects with alternating black and white vertical stripes. **(D)** illustrates a quadrant with a horizontal pattern of alternating black and white squares.

Sites were established at 10 km intervals from the southeast to the northwest of the Gurbantunggut Desert. Each site was divided into five 10 m × 10 m plots (**Figure 1**). In GIS, divide the east-west and north-south main roads of the Gurbantunggut Desert into grids, randomly select one grid as the starting point, and avoid obvious human interference areas (oil wells, 500 meter buffer zones on highways). Starting from a random starting point, set up one main sampling point every 10 km, for a total of 74 points. Within a range of 100 m × 100 m for each main sampling point, five 10 m × 10 m subplots are set using the system grid method (with 10 m intervals) to ensure spatial representativeness. Each plot was established in a typical interdune area which is flat with no slope. In this study, five 1 m × 10 m transect was established in a subplot. The transect was divided into 10-square grid plots with the aim of biocrusts type, coverage, distribution and patch size more conveniently and precisely (**Figure 1**). Within each 1 m × 1 m grid, first use a 30 cm × 30 cm sub grid to determine the patches with a biological crust coverage of ≥ 30%. All subsequent soil samples are strictly located directly below the patch and do not deviate laterally. Soil samples were selected from expose area with a minimum distance of 50 cm from surrounding shrubs. Soil samples were randomly collected within subplots. One soil sample in each subplot, which is a mixture of five randomly selected samples within the plot. If there is no biocrusts distribution, bare sand was collected. To precisely compare the properties of desert surface soils, we uniformly collected soil samples from 0-5cm of desert surface soil using a 5 cm height and diameter cutting ring. In total, 370 soil samples were collected from 74 sites, including 300 soils with biocrusts (80 alga-lichen and 220 moss) and 70 bare sand samples (**Figure 1**). For soil of biocrusts patches, we also collected the top 0–5 cm of soil. However, after collection, we removed the plant components from the biocrust’s soil samples, retaining only the soil part for analysis. The samples were collected in August 2018. Firstly, soil sand content was measured by weighing 50 g of dry soil, crushing, and passing through sieves with different mesh diameters to obtain soil particles of different sizes. The different soil particles were weighted and calculated the weight ratio. Soil samples were stored in a cool, dry place at an average room temperature of 25°C for 2 weeks, until the soil was dry.

### 2.3 Soil analysis

Use ASTM E11 standard copper sieve, oscillating sieve (Retsch AS200) with an amplitude of 1.5 mm and a screening time of 10 minutes. The sieve apertures (mm) are 2.0, 1.0, 0.5, 0.25, 0.15, and 0.075, respectively. Weigh them step by step to obtain the distribution of gravel and sand particles with an accuracy of 0.01 g, which are larger than 2 mm. A calibrated pH meter (PHS-4, Jiangsu Manufactory of Electrical Analysis Instruments, Jiangyin, China) was used to measure soil pH in a 1:5 soil:water suspension. DDS-11A (LEIC, Shanghai, China) was used to measure electrical conductivity (EC). Soil nutrient levels, i.e., organic carbon (SOC), total nitrogen (TN), total phosphorus (TP), total potassium (TK), NO_3_ + NO_2_-N (NO_3_-N), NH_4_-N, available phosphorus (AP), and available potassium (AK) were determined (Li et al., 2019). The dichromate oxidation method was performed to determine SOC contents. Soil organic carbon uses Chinese national standard substance GBW-07414 (standard value 1.05 ± 0.04%), with a recovery rate of 97-103%. soil TN was estimated by the Kjeldahl procedure after digestion with concentrated H_2_SO_4_ on a distillation unit, and TP was determined by the H_2_SO_4_ ammonium molybdate-ascorbic acid method (Barrett et al., 2007). TN and TP use GBW-07415, with a recovery rate of 96-104%. TK was quantified by using inductively coupled plasma spectrometry (Perkin Elmer Optima 3000-DV ICP, Perkin Elmer Inc., Shelton, Connecticut, USA). In order to obtain extractable inorganic nitrogen (NO_3_ + NO_2_-N and NH_4_-N), 10 g soil was extracted in 50 mL 2 M KCl, filtered, and frozen until run on a Lachat auto-analyzer (Barrett et al., 2007). For the analysis of the AP content, we used the molybdenum-antimony colorimetric method (Li et al., 2019). To measure the AK, 5 g soil was extracted in 50 mL 1 mol/L NH_4_Ac, filtered, and then frozen until run on an atomic absorption spectrophotometer (Perkin Elmer model 2380, Perkin Elmer Inc., USA).

Urease activity (µmol PNP g⁻¹ h⁻¹) was measured by the buffered hydrolysis reaction method modified by Li et al. (2019). In alkaline environments, soil alkaline phosphatase catalyzes the production of yellow p-nitrophenol from p-nitrophenyl phosphate, which has a maximum absorption peak at 405 nm. By detecting the rate of increase of PNP at 405 nm, the activity (µmol PNP g⁻¹ h⁻¹) of the alkaline phosphatase enzyme can be obtained. soil β-glucosidase can catalyze p-nitrobenzene-β-D-glucopyranoside generates a yellow substance, p-nitrophenol (PNP), which has characteristic light absorption at 405 nm, thereby obtaining the activity (µmol PNP g⁻¹ h⁻¹) of soil β-glucosidase (Li et al., 2019). Three technical repetitions were performed for each sample, RSD< 5%. β - glucosidase: LOD = 0.004 µmol PNP g^-1^ h^-1^, LOQ = 0.012 µmol PNP g^-1^ h^-1^; Alkaline phosphatase: LOD = 0.005 µmol PNP g^-1^ h^-1^, LOQ = 0.015 µmol PNP g^-1^ h^-1^; All measured values are>LOQ (**Supplementary Table S2**).

### 2.4 Soil multifunctionality

We estimated soil SMF using crucial functional variables, including soil SOC, TN, TP, TK, NH4-N, NO3-N, AP, AK, soil carbon, nitrogen, and phosphorus cycling enzymes. The selected functions are closely associated with soil fertility and soil nutrient cycling. They are commonly used as indicators to estimate soil nutrient cycling and soil fertility in drylands. Individual functions were standardized using the min-max transformation with a range from 0 to 1 (Equation 1). The minimum and maximum values of a soil function were estimated for the 74 studied sites.

(1) $$\begin{array}{c} f\left({\text{X}}_{\text{i}}\right)=\left({\text{X}}_{\text{i}}-\text{min}\left(\text{X}\right)\right)/\left(\max\left(\text{X}\right)-\text{min}\left(\text{X}\right)\right) \end{array}$$

where ${\text{X}}_{i}$ represents the value observed for the functional variables, min(X) denotes the minimum value, and max(X) denotes the maximum value observed. The index’s range is transformed to 0–1 through data normalization. Then, the soil SMF was calculated using the “averaging approach” (Equation 2) which is widely used in multifunctionality studies (Bowker et al., 2013; Su et al., 2021). The formula for calculating the P-SMF is presented as follows:

(2) $$\begin{array}{c} \text{SM}{\text{F}}_{\text{i}}=\frac{1}{\text{N}}\sum_{\text{i}=\;1}^{\text{N}}{\text{f}}_{\text{i}} \end{array}$$

In this equation, ${\text{SMF}}_{i}$ indicates the multifunctionality for sample i. N represents the overall functional variables count integrated into the computation, while ${\text{f}}_{i}$ denotes the standardized index of the functional variables for sample i. The arithmetic mean SMF was double checked by PCA weighting and threshold method, and the effect direction and significance were both robust.

### 2.5 Soil quality index

A scoring function analysis framework was used to calculate the SQI (Andrews et al., 2004; Karlen et al., 2008). The SQI was assessed by following a three-step procedure: (1) identification of the minimum dataset of indicators, (2) indicator interpretation, and (3) integration of all indicator scores into one overall SQI value (Andrews et al., 2004; Wang et al., 2024). Principal component analysis was performed on the standardized data matrix of the total dataset to determine potential soil indicators representing the minimum data set. Select principal components with eigenvalue > 1 and principal components with a total variation of at least 5% in the interpretation data set for minimum dataset identification. For each principal components, only the high load index whose load value is within 10% of the maximum weighted load is retained as an important index for indexing principal components.

Kaiser-Meyer-Olkin and Bartlett’s tests were used to determine whether the data were appropriate for principal component analysis. The Kaiser-Meyer-Olkin test was used to assess the correlation between the input variables. If the value of the Bartlett’s tests is significant (p< 0.05), it shows the correlation between the variables and the appropriateness of principal component analysis. In this study, the Kaiser-Meyer-Olkin statistic was > 0.6, and the BTS test was significant. In the minimum dataset, each highly loaded indicator was retained if they were not correlated. Otherwise, only the indicator with the highest weighted loading was chosen for the minimum dataset. After defining the minimum dataset for the SQI, each soil indicator was transformed into unitless combinable scores varying from 0.00 to 1.00 using linear and nonlinear scoring function methods (Andrews et al., 2004, Andrews et al., 2002; Raiesi, 2017). The appropriate scoring algorithms for “scoring indicators” values were selected and interpreted for soil productivity and sustainability. “More is better” and “less is better” scoring curves were used to indicators when a soil indicator was considered good for SQI in increasing order (more is better) or in decreasing order (less is better). For linear scoring, “more is better” (Equation 3) or “less is better” (Equation 4) functions were used as follows:

(3) $$\text{Si}=0.1+\left(\frac{\left(\text{x}-\text{b}\right)}{\left(\text{a}-\text{b}\right)}\right)\times 0.9$$

(4) $$\text{Si}=1-\left(\frac{\left(\text{x}-\text{b}\right)}{\left(\text{a}-\text{b}\right)}\right)\times 0.9$$

Where Si was the transformed x variable, b and a are the minimum and maximum threshold values of the x (non-transformed or true variable), respectively.

Nonlinear scoring functions (NLSF), proposed by Masto et al. (2008), were used to normalize SQI (Equation 5)as below:

(5) $$\text{Si}=\frac{1}{\left[1+{\text{e}}^{-\text{β}\left(\text{x}-\text{α}\right)}\right]}$$

Where a is the baseline value of the soil variable, where the score is 0.5 or close to the mean value of the upper and lower thresholds and b is the slope. Baselines are generally considered the lowest target values. An acceptable condition can be considered for the system if the soil indicator value is within the threshold (control) limits. If the value is not within the control limits, it is considered a degraded system (Masto et al., 2008).

After scoring the selected indicators, we applied a weighted additive approach to integrate them into the indices (Andrews et al., 2002). In this study, the weights (Wi) of the minimum dataset indicators were given by the ratio of the variance of each variable to the total cumulative variance to get a certain weight value under principal component analysis (Tesfahunegn, 2014). The scoring was calculated using the “more is better” method in this study. After weighing the scored minimum dataset indicators, the integrated SQI was calculated using the below equation (Qi et al., 2009):

(6) $$\text{SQI}=\sum_{i=1}^{n}WiSi$$

where Wi and Si are the weight and score of indicators i, respectively.

Perform PCA (KMO = 0.74, Bartlett p<0.001) after standardizing the 12 original indicators. Retain the first four principal components based on eigenvalues>1, and explain 83.1% of the variance cumulatively. If Pearson | r |>0.70 for both indicators, only retain the one with the highest load (SOC and TN r=0.71, retain SOC); The final minimum dataset (MDS) contains 7 indicators: SOC, TP, AK, ALP, BG, pH, and EC. The final SQI (0-1) is obtained by weighting and summing the variance contribution rate of each indicator’s PC to the cumulative variance ratio. After changing from non-linear to linear scoring, the SQI (Equation 6) and original results have r=0.93, and the direction of the effect remains unchanged. Delete any indicator, and if the site ranking Kendall τ>0.89, it indicates that the weight and function selection are robust.

### 2.6 Spatial variation of soil multifunctional

SVM is estimated by first calculating the variability of the soil variables measured at each site. To do so, we estimated the site-level coefficient of variation (CV) using the composite soil samples collected at each site. The CV is a relative measure of heterogeneity that can accommodate variance-mean scaling, preventing variances from increasing with the mean. Therefore, it is more useful for comparing variability within biological properties than absolute measures of variability, such as standard deviation (Fraterrigo and Rusak, 2010; Schlesinger et al., 1990). We estimated SVM (Equation 7) as the arithmetic mean for all individual site-level CVs of all soil variables.

(7) $$\begin{array}{c} \text{SV}{\text{M}}_{\text{i}}=\frac{1}{\text{N}}\sum_{\text{j}=\;1}^{\text{N}}\text{C}{\text{V}}_{\text{j}} \end{array}$$

where ${\text{CV}}_{j}$ represents the value of CV for the functional variables in a site. Then, the soil SVM was calculated using the “averaging approach” which is widely used. SVMi indicates the SVM for site i. N represents the overall functional variables count integrated into the computation.

The latitude, longitude, and altitude data for the sites were acquired using a GPS locator. The environmental site information was sourced from the National Meteorological Science Data Center (http://data.cma.cn/site/index.html) and the Resource and Environmental Science Data Center of the Chinese Academy of Sciences (http://www.resdc.cn) and included variables such as the MAP, MAW, radiation (Rad) and MAT. The data source has a spatial resolution of 0.25° × 0.25° (approximately 25 km) and a 30-year climate standard value spanning from 1981 to 2010. Using ArcGIS 10.8, perform bilinear interpolation between the GPS coordinates (WGS84) of 74 sampling points and the nearest grid point to obtain the station level annual average; Interpolation error ≤ 0.3°C (MAT) and ≤ 3 mm (MAP), meeting the requirements of regional scale research.

### 2.7 Statistical analyses

We used R 4.1 software to perform all statistical analyses. We used linear mixed-effects models to account for the hierarchical structure (Plot nested within Site) when testing the effect of biocrust type on SMF, SQI and SVM. Here, we calculated the synthesized biocrusts index following the same process as SMF using biocrusts type, thickness, cover and patch characteristics, which are variables that have been used to indicate biocrust type development and characteristics in previous studies (Belnap et al., 2008; Chamizo et al., 2012; Kidron et al., 2010; Lan et al., 2013). The biocrusts index is closely correlated with visual biocrusts type development (Su et al., 2021). We explored the relationships between MAT, MAP, MAW using regression analysis to evaluate the effects of abiotic factors on biocrusts index. We explored the relationships between biocrusts index, MAT, MAP, MAW, soil pH and EC using regression analysis to evaluate the effects of biotic and abiotic factors on soil SMF, SQI and SVM. Only when non-linear regressions were a better fit to the data, thresholds may be present. Collinearity was absent (VIF< 3.2); MAP × MAT interaction and GAMM non-linearity confirmed the 163 mm threshold without significant interaction terms. Therefore, we explored the presence of thresholds only when non-linear models were a better fit to the data. We did so because segmented regressions threshold models force the existence of at least one threshold. The regression analysis of MAP and SMF, MAP and SQI used segmented linear regression to find the threshold and then conducted linear regression analysis. The breakpoint was estimated by segmented regression with 9999 bootstrap replicates, yielding 163 mm (95% CI = 154 - 172).The regression analysis of soil pH with SMF and SQI used segmented linear regression to find the threshold and then performed linear regression analysis.

In this study, we employed structural equation modeling (SEM) to investigate the direct and indirect effects of biotic and abiotic drivers on the SMF, SQI and SVM. Firstly, we establish an initial model. Subsequently, the model was revised based on the initial model to achieve the optimal model. The following criteria were used to evaluate the model fit: Fisher’s p value (model fits well at 0.05< p< 1.00) and Akaike’s information criterion (AIC; model fits well at low AIC). Spatial Moran’s I and d-separation tests showed no residual autocorrelation (p > 0.35), supporting the independence assumption of SEM. Other unnecessary pathways were eliminated, and only meaningful pathways were retained in the final model. To construct the SEM and gain a comprehensive understanding of the primary drivers of the SVM, we utilized the “piecewiseSEM” package.

## 3 Results

### 3.1 SMF, SQI and SVM of biocrusts

**Figure 2:**
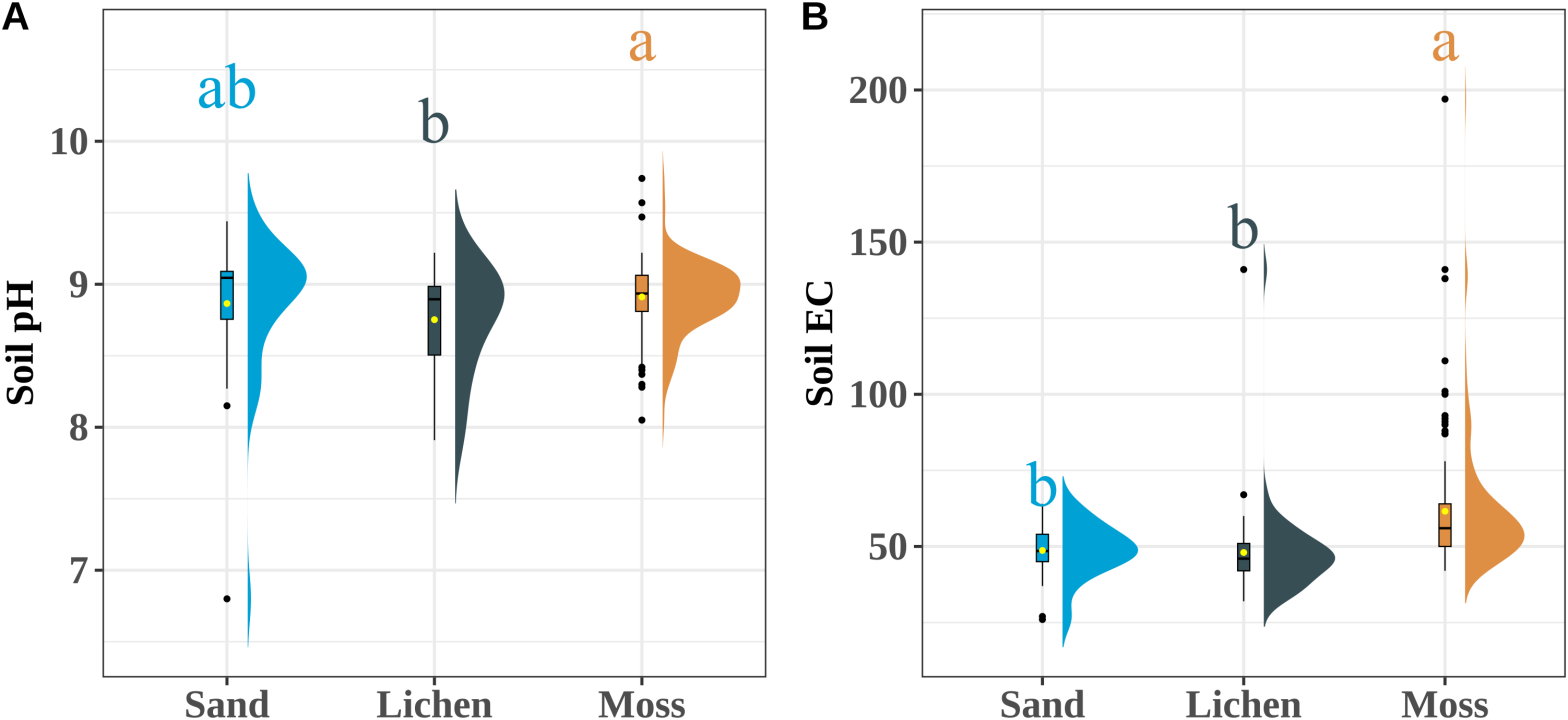
Changes of soil pH **(A)** and EC **(B)** in interspaces of different BSC patches (Sand: bare sand, Lich: lichen crust, Moss: moss crust). Different letters (a, b) above the box plot indicate significant differences at *p* < 0.05. The sample size of sandy soil is 70, the sample size of algae and lichen crusts is 80, and the sample size of moss crusts is 220.

**Figure 3:**
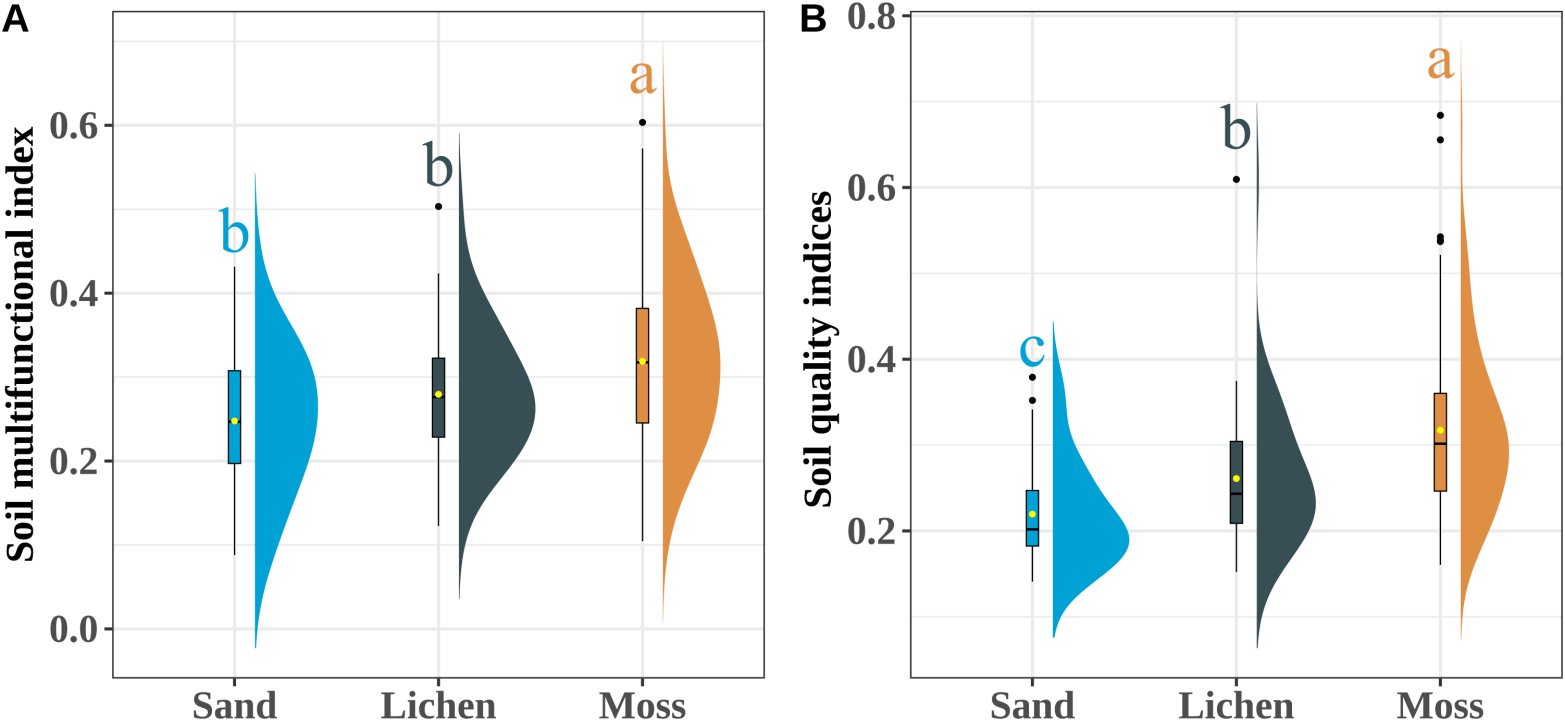
Changes of SMF **(A)** and SQI **(B)** in interspaces of different BSC patches (Sand: bare sand, Lich: lichen crust, Moss: moss crust). Different letters (a–c) above the box plot indicate significant differences at *p* < 0.05. The sample size of sandy soil is 70, the sample size of algae and lichen crusts is 80, and the sample size of moss crusts is 220.

Soil under moss crusts exhibited the highest soil SMF and SQI compared with bare sand and algae-lichen crusts (**Figure 2**). The SQI of soil beneath algae-lichen crusts was significantly higher (*p* < 0.05) than that of sand. There was no significant difference between sand and algae-lichen crusts. Surface soil TN, available nitrogen (NO_3_-N and NH_4_-N), AP, AK, pH, and EC differed significantly among biocrust types (*p* < 0.05) (**Supplementary Figures S5**–**S7**). The biocrust index increased with rising MAT (*p* = 0.003, R^2^ = 0.12) and MAW (*p* < 0.001, R^2^ = 0.29) (**Figure 3**). The biocrust index rose with MAP up to 163 mm (*p* = 0.007, R^2^ = 0.26) and then declined (*p* = 0.002, R^2^ = 0.18)(**Figure 3**).

### 3.2 The influence of climate and biocrusts

**Figure 4:**
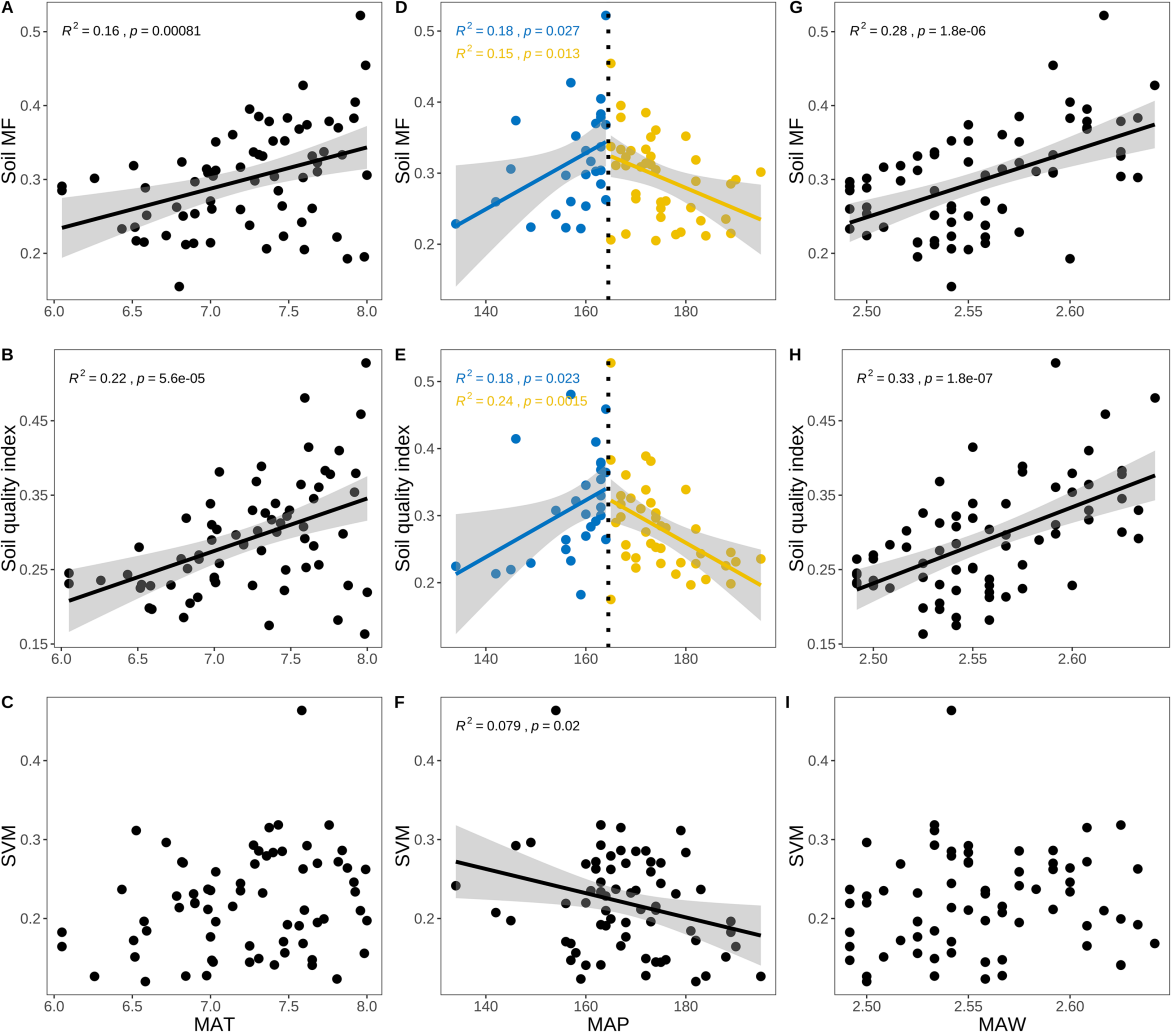
The trend of soil SMF, SQI and SVM with MAT, MAP and MAW increased. **(A–C)** The changes of SMF, SQI and SVM with MAT increased; **(D–F)** The changes of SMF, SQI, SVM with MAP increased; **(G–I)** The changes of SMF, SQI, SVM with MAW increased. Equations on each plot show the p and R^2^ of linear regression. The total sample size is 370.

**Figure 5:**
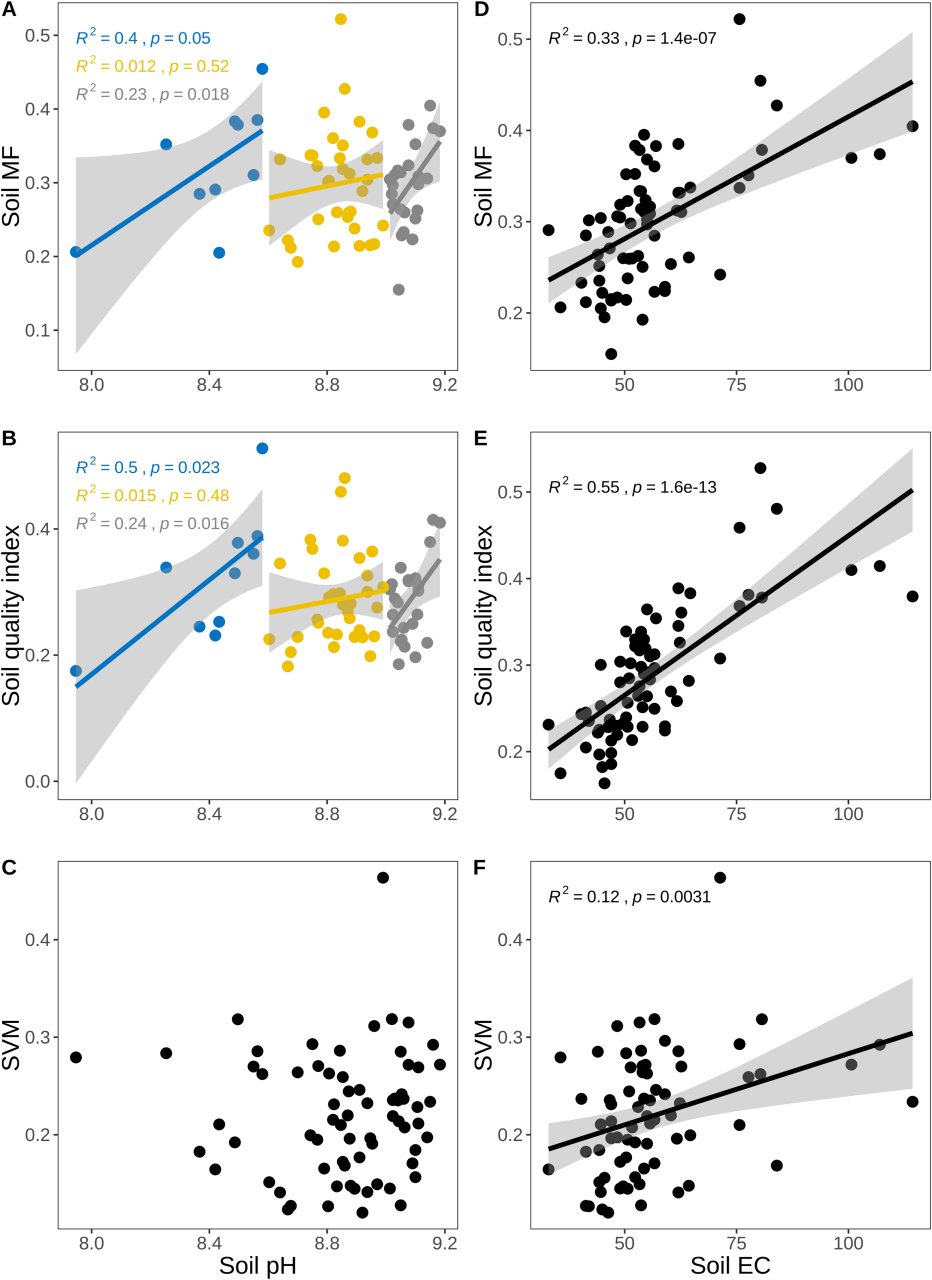
The trend of soil SMF, SQI and SVM with soil pH and EC increased. **(A–C)** The changes of SMF, SQI and SVM with soil pH increased: **(D–F)** The changes of SMF, SQI, SVM with soil EC increased. Equations on each plot show the p and R^2^ of linear regression. The total sample size is 370.

The SMF (*p* < 0.001, R^2^ = 0.47) and SQI (*p* < 0.001, R^2^ = 0.62)increased with an rising biocrusts index (**Figure 4**). There was no obvious change in SVM with increasing biocrusts index. SMF and SQI rose with increasing MAP up to a peak and then declined (**Figure 5**). Both metrics were initially positively correlated with MAP (SMF: *p* = 0.027, R^2^ = 0.18; SQI: *p* = 0.023, R^2^ = 0.18), and both decreased when MAP > 163 mm (SMF: *p* = 0.013, R^2^ = 0.15; SQI: *p* = 0.0015, R^2^ = 0.24). SVM and MAP were negatively correlated (*p* = 0.02, R^2^ = 0.079). SMF and SQI also increased with MAT (SMF: *p* < 0.001, R^2^ = 0.16; SQI: *p* < 0.001, R^2^ = 0.22) and with MAW (SMF: *p* < 0.001, R^2^ = 0.28; SQI: *p* < 0.001, R^2^ = 0.33), whereas SVM showed no significant change with MAT (*p* > 0.05) or MAW (*p* > 0.05) (**Figure 5**).

**Figure 6:**
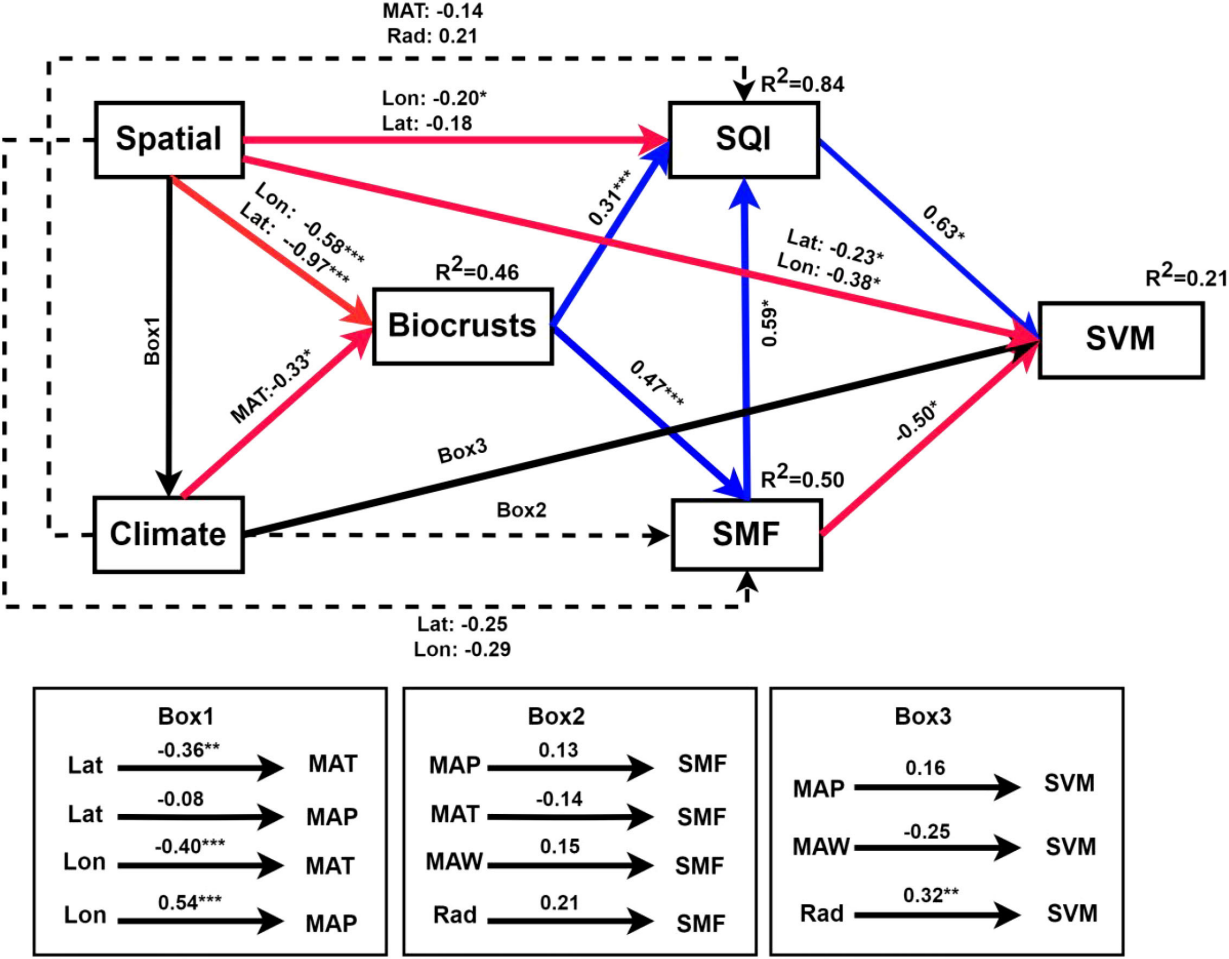
Final fitted structural equation models representing relative effects of climate (MAT, MAP, MAW, Rad), geographical position (Lat, Lon), BSC (BSC indicate), soil pH, soil EC, soil sand content (Sand), soil SMF, SQI, and SVM. Boxes signify measured variables. Spatial: latitude (Lat) and longitude (Lon); Climate: include MAT, MAP, MAW, Rad. Standardized path coefficients are displayed, with the width of each arrow equivalent to the strength of the path. Red lines denote the negative paths (*p* < 0.05). Blue lines indicate positive paths (*p* < 0.05). The total amount of variance (R^2^) explained for each endogenous variable (those with arrows pointing to them) is given below the variable. Corresponding probability values are included when p< 0.05 (*p < 0.05, **p < 0.01, ***p < 0.001). The fit of the model was statistically tested (Fisheries’C = 39.833, df = 62, *p* = 0.987).

The SEM results showed that the model accounted for 21% of the variance in SVM (**Figure 6**). Spatial coordinates strongly influenced biocrusts (Lon: –0.58, Lat: –0.97), SQI (Lon: –0.20, Lat: –0.18) and SVM (Lon: –0.38, Lat: –0.23). MAT exerted a significant negative effect on biocrust development and distribution (-0.33). Biocrusts had significant positive effects on soil SMF (0.47) and SQI (0.31). Biocrusts did not significantly affect SVM. Soil SMF had a significant negative effect on SVM (- 0.50), whereas SQI had a significant positive effect on SVM (0.63).

## 4 Discussion

### 4.1 Effect of biocrust types on SMF and SQI

Our findings confirm that biocrust type significantly modulates both SQI and SMF. Compared with algal–lichen crusts, moss-crusted soils exhibited markedly higher TN, NO_3_-N, NH_4_-N, AP, AK, pH, EC, SQI and SMF. Previous studies have demonstrated that as the biological crust layer transitions from algal-lichen crust to moss crust, the soil nutrients beneath the biological crust exhibit an increase (Gao et al., 2018; Fan et al., 2021). Thus, our results align with previous studies showing that biocrusts strongly modulate soil nutrients and physical properties within their colonized zones. One reason for this result is that the distribution of biocrusts in desert ecosystems stabilize the soil (Weber et al., 2022; Yang et al., 2022); The increase in soil stability could also lead to an increase in SMF and SQI. Furthermore, biocrusts play a critical role in regulating soil moisture dynamics by enhancing atmospheric water vapor condensation, decreasing evaporation, boosting water retention, and stabilizing soil surfaces. These effects are particularly pronounced in arid and semi-arid ecosystems, where biocrusts can markedly increase soil water retention and availability for plant growth (Li and Giora, 2021; Li and Xiao, 2022; Sun et al., 2021; Xiao et al., 2022, Xiao et al., 2019). The “fertilizer island” effect exhibited by biocrusts can improve nutrient availability and thus improve soil function (Agnelli et al., 2021; Li et al., 2019; Pushkareva et al., 2017; Young et al., 2022). Furthermore, the “fertilizer island” effect of biocrusts contributes to the accumulation of soil nutrients, which may cause nutrient accumulation of desert surface soil and increase the SMF and SQI.

Biocrusts are well-known for driving the nutrient cycle of desert soil (Wang et al., 2021; Young et al., 2022). Algae, lichens and mosses can increase soil stability by enhancing soil aggregation and reducing erosion (Lange and Belnap, 2016). Therefore, we suggest that the changes in soil SMF and SQI were due to the biocrusts type. Chamizo et al. (2017) validated that biocrusts removal enhanced sediment yield by 20 to 60 times more than that of undisturbed soils colonized by lichen. By reducing biocrust, desert ecosystems may experience a reduction in soil carbon, nitrogen fixation and phosphorus activation and mineralization (Gao et al., 2020). The soil SMF and SQI in moss crust were considerably higher than those in algae-lichen crust and sand. Moss crust, has high soil stability and nutrient accumulation, and can enhance soil nutrients, SMF and SQI in moss crust patches. Hence, we consider that biocrusts can influence SMF and SQI, but this effect is moderated by the biocrusts type and mostly occurs in moss crust.

### 4.2 Soil SMF, SVM, and SQI are regulated by climate and biocrusts

Desert ecosystems are extremely sensitive to climate change, therefore climate can significantly affect the vegetation distribution characteristics in desert ecosystems. Our results confirm this conclusion that climate change can significantly affect the distribution and developmental characteristics of biocrusts. The biocrusts index shows a significant increasing trend with the increase of MAT and MAW. Previous research has found that temperature have a significant impact on the development of biocrusts type (Ferrenberg et al., 2015). Moreover, the SMF, SQI and SVM increase with the increase of MAT. The apparent contradiction arises because simple regression captures the net temperature benefit, whereas SEM partitions it into opposing direct (negative) and indirect (positive) components. Temperature affects the microbial activity and metabolic processes in biocrusts, and a suitable temperature is beneficial for the transformation from algal-lichen crust to moss crust (Zelikova et al., 2012). Changes in temperature can alter soil moisture content, thereby affecting soil aeration, permeability, and cohesion (Li et al., 2018; Wei et al., 2022). Temperature changes can affect the rate of chemical reactions in soil, such as the release and fixation of mineral nutrients in soil. After accounting for other variables, warming exerts a direct inhibitory effect on microbial activity-primarily by accelerating evaporation and reducing soil water potential-which consequently diminishes SMF. At the same time, warming also indirectly enhances SMF through two mechanisms: prolonging the growing season and improving the utilization of condensed water, thereby promoting moss crust development. These opposing mechanisms operate simultaneously within the same plots and across the same climate gradient. Their net effect remains weakly positive due to partial cancellation of positive and negative contributions, which explains why simple regression and SEM both indicate an overall positive influence of warming on SMF, despite exhibiting opposing signs in their direct pathways. Under different temperature conditions, the types and quantities of organisms in the soil will vary, thereby affecting the SMF. This shift contributes to an increase in soil moisture retention, SQI, and soil stability.

Precipitation is an important environmental factor in desert ecosystems. Precipitation provides the necessary water for microorganisms in biocrusts, and usually the more precipitation, the better the development of different biocrusts types (Wu and Zhang, 2018). Therefore, biocrusts type affects SMF and SQI both directly and indirectly across a climatic gradient (Su et al., 2021). It directly alters SMF and SQI by increasing soil carbon and nitrogen fixation, and phosphorus desorption (Bowker et al., 2014; Xu et al., 2021). Moreover, the aggregation and growth of photoautotrophic organisms in surface soil provide effective coverage and soil carbon storage in areas where plant growth is limited owing to sporadic precipitation (Lefcheck et al., 2015; Maier et al., 2018). Biocrusts type development and the accompanying functional traits also indirectly affect SMF and SQI by altering the community abundance and composition of soil microbial communities (Garibotti et al., 2018; Su et al., 2021; Zhou et al., 2023).

We found that climate directly influenced the soil SMF and SQI. The SMF and SQI show a one-peak model with the increase of MAP. The SMF and SQI are the highest when the MAP is 163 mm (**Figures 3**, **5**). The moss crusts increase with the increase of MAP in the desert. Simultaneously, some studies found that the relative positive effects of biocrust-forming mosses on multifunctionality compared with bare soil increased with increasing aridity, and that biocrusts is crucial to buffer negative effects of climate change on multifunctionality in global drylands (Delgado-Baquerizo et al., 2016). Precipitation affects soil moisture and permeability. Moderate water content helps dissolve and transport soil nutrients, but excessive precipitation may lead to soil erosion (Currier and Sala, 2022). The increase in precipitation may increase the leaching and reduce the nutrient concentration on the soil surface. The present study found that with the increase in precipitation, the microbial community structure of desert soil also increased first and then decreased, and the nutrient content of moss also increased. Furthermore, soil microbial communities and biocrusts species could regulate the effects of global change on SMF in drylands (Liu et al., 2017). All water inputs (rainfall, snow-melt, dew) ultimately derive from precipitation, so we treat them collectively as the precipitation driver. Below the 163 mm threshold every extra 1 mm of MAP increases SMF by 0.8% and SQI by 0.6% (**Figure 4**) because more frequent wet pulses prolong biocrust photosynthesis. Therefore, the response of the SMF and SQI to MAP is a complex process in drylands. This complexity is further compounded by the interactions between various biotic and abiotic factors. The divergence point (where SQI plateaus but SVM continues to rise) coincides with our 163 mm MAP threshold; future work should test whether this decoupling is universal across temperate deserts.

### 4.3 SMF, SQI is mainly driven by biocrusts, but is influenced by climate

Based on the results of the SEM, it can be concluded that SQI is mainly affected by the biocrusts and soil SMF. There is a significant negative effect of soil SMF on SVM, but a significant positive effect on SQI. Moreover, it is also found that the Lat and Lon of soil samples and climate can markedly influence biocrusts, SMF and SQI. Due to different soil substrates, SMF and SVM are significantly different between regions. In line with the previous studies, SMF and SQI are affected not only by the biocrusts but also by the climate (Ferrenberg et al., 2022; Huang et al., 2020; Hui et al., 2022; Xiao and Bowker, 2020). Biocrusts have been shown to play an essential role in controlling ecosystem responses to climate change (Maestre et al., 2013; Reed et al., 2012). For example, temperature, soil moisture, and nutrient limitation are thought to affect soil carbon metabolisms, such as microbial growth, respiration, carbon use efficiency, and microbial biomass turnover (Takriti et al., 2018; Hagerty et al., 2014; Dijkstra et al., 2015; Schindlbacher et al., 2015; Zheng et al., 2019). Temperature, soil pH, enzyme activity, and substrate quality can influence soil nutrient cycling (Noll et al., 2019; Wallenstein and Weintraub, 2008; Wanek et al., 2011). Therefore, the results demonstrate that SMF and SQI are substantially influenced by biotic factors (biocrusts type) and abiotic factors (climate). We consider that the spatial and climate in the desert ecosystem are the significant factors that directly affect the SMF, SQI, and SVM.

In this study, we focused on the impact of different types of biocrusts on soil SMF, SQI and SVM in the Gurbantunggut Desert. However, there are certain limitations to this study, as we concentrated solely on two types of biocrusts in the Gurbantunggut Desert. This study has several limitations. First, the findings are based on only two types of biocrusts (algal-lichen and moss crusts) within a single desert region, which may constrain the broader applicability of the results. Second, as biocrusts globally encompass a wider variety of forms, including pure algal, lichen, moss, and mixed assemblages, the functional diversity captured in this study may not be fully representative. Third, the lack of data on associated vegetation and soil microbial communities limits our understanding of their interactive effects with biocrusts on soil multifunctionality and quality.

Biocrusts represent some of the earliest pioneer species in vegetation succession, characterized by minimal resource consumption, and they hold significant potential for applications in desertification control, ecological conservation, and restoration. In the desert regions of northwest China, biocrusts exhibit extensive distribution, high species diversity, and a rich variety of types. However, comprehensive and comparative research on the diversity of biocrusts across different desert areas is lacking, as is a systematic understanding of their relationship with soil nutrient functions. Therefore, in the future, we will primarily investigate the composition and diversity of spore plants and microorganisms in various biocrust types in desert regions, particularly under the influence of global climate change, as well as their relationship with soil functions and quality. Moreover, we aim to enhance our understanding of the relationship between soil nutrient cycling characteristics and the physical and chemical properties of biocrusts, along with the sensitivity of different crust types to climatic factors. We will further explore the application of biocrusts and propose new insights into ecological restoration practices in arid regions, thereby providing valuable decision-making support for the management of desert ecosystems and the restoration of degraded ecosystems.

These limitations highlight the need for future studies to expand the scope of biocrust types examined, including pure algal crusts, cyanobacterial crusts, and mixed crustal assemblages, across multiple desert ecosystems with varying climatic conditions and soil substrates. Establish a synchronous observation network in temperate deserts (e.g., Taklamakan and Badain Jaran), integrating micrometeorological measurements and unmanned aerial vehicle remote sensing to build a dynamic, long-term (≥10 years) dataset on soil multifunctionality in biological crusts. This will help validate the universality of the 163 mm precipitation threshold identified in this study. Using metagenomics and ^15^N/^13^C isotope labeling techniques, we will quantify the expression levels of key functional genes (nifH, amoA, phoD) involved in carbon fixation, nitrogen fixation, and phosphorus mineralization across different crust types. This approach aims to clarify the microbial mechanisms through which biological crusts drive soil multifunctionality (SMF). Along the natural climate gradient on the eastern edge of the desert, field manipulation platforms will be deployed to simulate warming (+2°C, +4°C) and altered precipitation regimes (± 30%), combined with crust type replacement treatments. These experiments will allow quantitative distinction between the direct effects of climate and the indirect effects mediated by shifts in crust type on SMF, soil quality index (SQI), and soil vulnerability mitigation (SVM). In subsequent studies, metagenomic sequencing and qPCR will be applied to compare the abundance and expression patterns of key functional genes (e.g., nifH, amoA, phoD) related to carbon fixation, nitrogen fixation, and phosphorus transformation in algal-lichen and moss crusts. A random forest model will be employed to quantify the relative contribution of microbial community structural changes to SMF components, thereby elucidating the microbiological mechanisms by which biocrust type regulates SMF.

## 5 Conclusions

The present study demonstrates that biocrusts type can significantly and directly affect the soil SMF and SQI, and can also indirectly affect SVM by altering SMF and SQI. Moreover, the climate can also indirectly affect SMF, SQI and SVM by altering biocrusts type. This reinforces the notion that biocrusts are major structural and functional components in drylands. Our results emphasizes that it is crucial to assess them in different biocrusts types and climates if we aim to better understand their role in driving ecosystem function in highly heterogeneous ecosystems such as drylands. We, for the first time, quantified a MAP threshold of 163 mm as the peak inflection point between moss crust cover and SMF. This threshold offers a scientific basis for defining the precipitation boundary and the upper limit of artificial precipitation in ecological restoration efforts across temperate deserts in Central Asia. In these ecosystems, the probability of natural moss crust establishment is highest in areas with MAP between 145–180 mm and a MAT of 6-8°C. It is therefore recommended that artificial moss crust inoculation be prioritized within this climatic range. Due to the lack of information on vegetation and soil microbial communities in this study, it is not possible to calculate the relationships between vegetation, biocrusts and soil microorganisms, as well as their impact on soil SMF, SQI and SVM. We suggest using biocrusts as a medium for soil aboveground and underground, and further exploring the structure and function of desert ecosystems by combining vegetation and soil microbial diversity and community structure. Our research findings contribute to understanding the distribution of different biocrusts types in desert ecosystems and their impact on soil nutrients, functions, and quality, providing a scientific basis for ecological restoration and sustainable management of desert ecosystems.

## Data Availability

The raw data supporting the conclusions of this article will be made available by the authors, without undue reservation.
