# Supplementary material for: Climate and biocrust types jointly regulate soil multifunctionality and quality in drylands: evidence from the Gurbantunggut Desert
Source: Front Plant Sci. 2026 Feb 10;17:1670208. doi: 10.3389/fpls.2026.1670208 (PMC12929415; doi:10.3389/fpls.2026.1670208)
Supplement: Supplementary file 1 [file Table1.docx]

Supporting Information

**Conceptual diagram of climate crust soil functional framework and hypothetical pathways**

Climate (temperature, precipitation, wind speed) points to BSCs, and climate drives changes in crust types and coverage.

These changes in turn affect the distribution and function of soil microbial communities. The interaction between climate factors and biological soil crusts plays a crucial role in maintaining ecosystem stability. By influencing the physical and chemical properties of the soil, these processes contribute to the overall health of the environment. Understanding these dynamics is essential for predicting how ecosystems will respond to future climate scenarios. BSCs drive changes in soil multifunctionality and soil quality. The complex interplay between biological soil crusts and various environmental factors creates a dynamic system that continuously shapes soil characteristics. These interactions not only influence immediate soil conditions but also establish long-term patterns that affect ecosystem resilience. The capacity of BSCs to modify their surroundings demonstrates a sophisticated adaptation mechanism that has developed over time. As these crusts interact with changing climate conditions, they reveal both vulnerabilities and remarkable adaptive potential within the ecosystem. This understanding highlights the need for comprehensive monitoring approaches to capture these intricate relationships accurately.

**
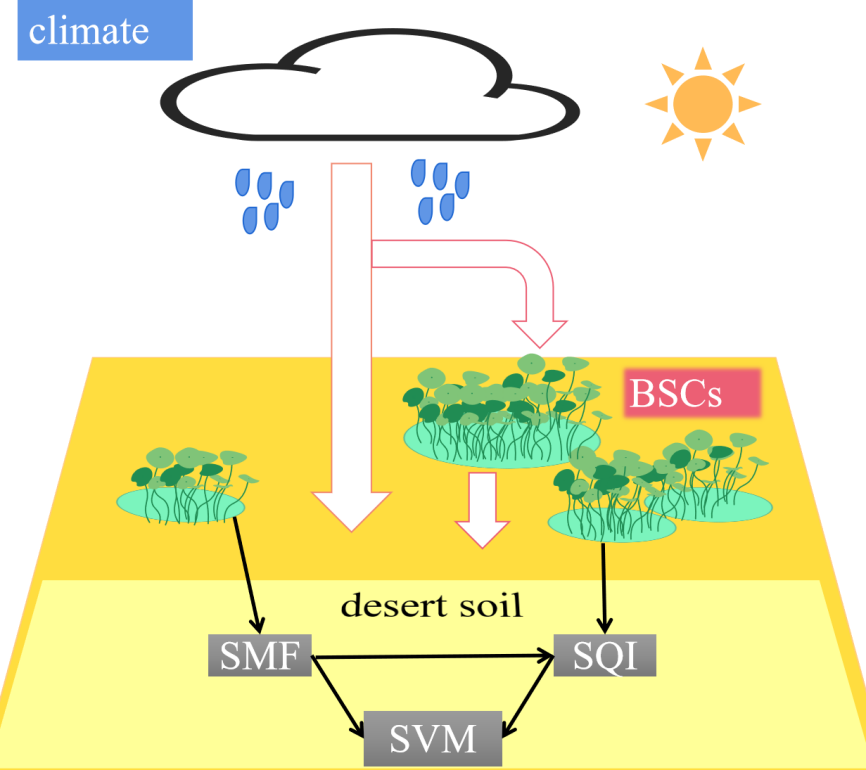
**

Fig S1. Conceptual diagram of climate crust soil functional framework and hypothetical pathways

**The landscape in different soil types**

The sites are conducted from southeast to northwest along the Gurbantunggut Desert. The landscape of each site is not the same, mainly including the undistributed biocrusts, algal crust with a small amount of lichen distribution, lichen and algal crust, and moss crust. The bare site is undistributed biocrusts. The black area in the bare site is coarse sand. There are relatively few algae and lichens distributed in algal and lichen site, and their coverage is very low. The biocrusts is mainly dominated by lichens in lichen site. The three types of sand, lichen and moss (sand: bare site, lichen: algal & lichen site and lichen site, moss: moss site), are used throughout the article (Fig S2). We can visually distinguish moss crusts from other crusts (algae lichen crusts) through observation in the wild (Fig S3). This visual distinction is further enhanced by the dense, carpet-like structure that moss forms, contrasting sharply with the looser arrangements typical of algae and lichen. In areas where moss dominates, the soil surface appears more cohesive and stable, reducing erosion risks significantly. Additionally, the presence of moss often indicates a higher level of soil moisture retention, as its structure helps trap water near the surface. These characteristics collectively contribute to the unique role moss plays in shaping the landscape across different soil types.

Fig S2. Spatial distribution map of precipitation and different biological soil crust types in the gurbantunggut desert.


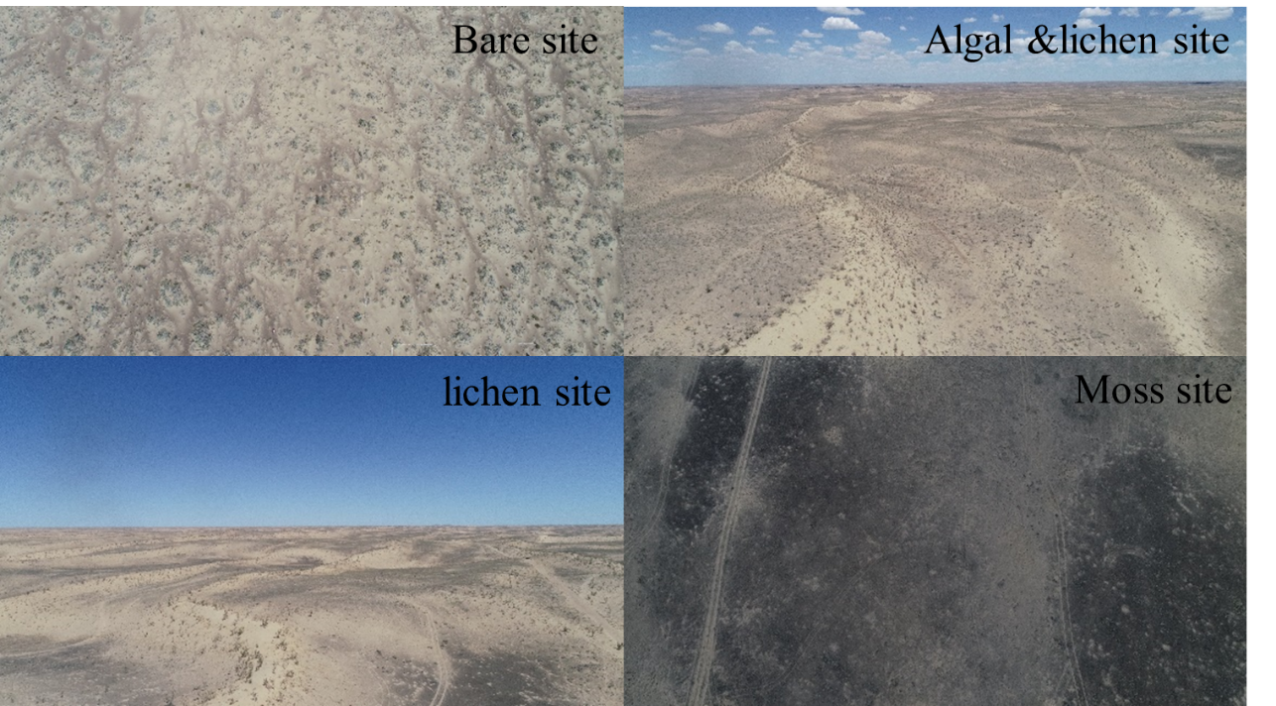


Fig S3. Landscape map of biological crust distribution in different sites.


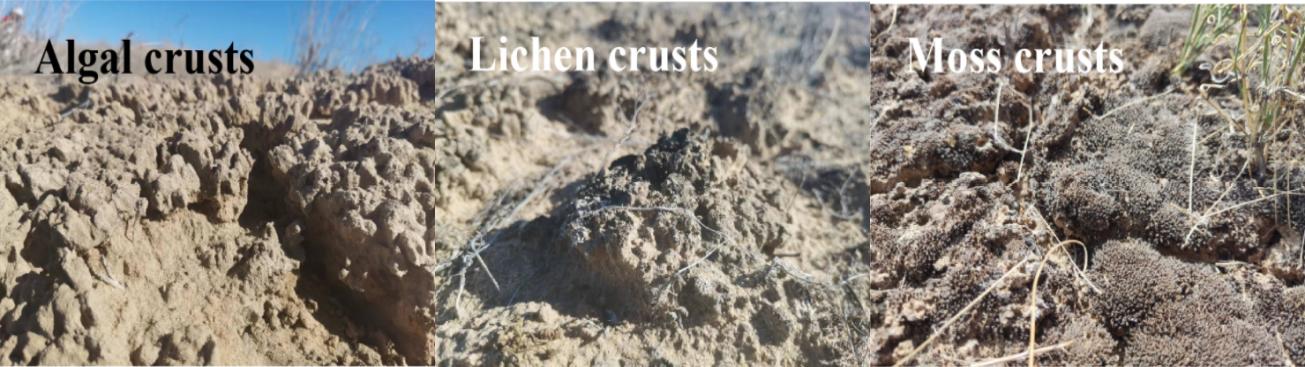


Fig S4. Photographs of different types of biological soil crusts.

**BSCs patch coverage**

Table S1 The number and coverage of biological crust patches.

| Site | Soil type | Algal and lichen | Moss |
| --- | --- | --- | --- |
| 1 | Algal and lichen | 0.646 |  |
| 2 | Moss | 0.066 | 0.648 |
| 3 | Moss | 0.1 | 0.647 |
| 4 | Moss | 0.103 | 0.635 |
| 5 | Moss | 0.095 | 0.642 |
| 6 | Moss | 0.093 | 0.645 |
| 7 | Moss | 0.101 | 0.639 |
| 8 | Algal and lichen | 0.685 |  |
| 9 | Algal and lichen | 0.6341667 |  |
| 10 | Algal and lichen | 0.6241667 |  |
| 11 | Moss | 0.105 | 0.529 |
| 12 | Moss | 0.064 | 0.447 |
| 13 | Moss | 0.101 | 0.48 |
| 14 | Moss | 0.082 | 0.574 |
| 15 | Moss | 0.068 | 0.607 |
| 16 | Moss | 0.083 | 0.362 |
| 17 | Moss | 0.068 | 0.462 |
| 18 | Moss | 0.07 | 0.487 |
| 19 | Moss | 0.077 | 0.473 |
| 20 | Moss | 0.063 | 0.391 |
| 21 | Moss | 0.05 | 0.557 |
| 22 | Moss | 0.067 | 0.537 |
| 23 | Moss | 0.058 | 0.36 |
| 24 | Moss | 0.106 | 0.474 |
| 25 | Moss | 0.074 | 0.563 |
| 26 | Algal and lichen | 0.443 |  |
| 27 | Algal and lichen | 0.539 |  |
| 28 | Algal and lichen | 0.461 |  |
| 29 | Algal and lichens | 0.605 |  |
| 30 | Algal and lichen | 0.502 |  |
| 31 | Algal and lichen | 0.498 |  |
| 32 | Algal and lichen | 0.569 |  |
| 33 | Algal and lichen | 0.447 |  |
| 34 | Algal and lichen | 0.429 |  |
| 35 | Moss | 0.089 | 0.394 |
| 36 | Moss | 0.067 | 0.339 |
| 37 | Moss | 0.052 | 0.588 |
| 38 | Moss | 0.073 | 0.397 |
| 39 | Algal and lichen | 0.566 |  |
| 40 | Sand |  |  |
| 41 | Algal and lichen | 0.607 |  |
| 42 | Moss | 0.061 | 0.577 |
| 43 | Moss | 0.052 | 0.546 |
| 44 | Moss | 0.109 | 0.423 |
| 45 | Algal and lichen | 0.607 |  |
| 46 | Algal and lichen | 0.538 |  |
| 47 | Moss | 0.099 | 0.595 |
| 48 | Moss | 0.105 | 0.522 |
| 49 | Moss | 0.108 | 0.595 |
| 50 | Moss | 0.067 | 0.524 |
| 51 | Moss | 0.051 | 0.607 |
| 52 | Sand |  |  |
| 53 | Algal and lichen | 0.062 |  |
| 54 | Moss | 0.089 | 0.538 |
| 55 | Sand |  |  |
| 56 | Sand |  |  |
| 57 | Sand |  |  |
| 58 | Moss | 0.058 | 0.584 |
| 59 | Moss | 0.106 | 0.416 |
| 60 | Moss | 0.074 | 0.55 |
| 61 | Moss | 0.088 | 0.428 |
| 62 | Moss | 0.074 | 0.352 |
| 63 | Moss | 0.094 | 0.302 |
| 64 | Moss | 0.106 | 0.496 |
| 65 | Moss | 0.05 | 0.351 |
| 66 | Moss | 0.102 | 0.41 |
| 67 | Algal and lichen | 0.416 |  |
| 68 | Sand |  |  |
| 69 | Sand |  |  |
| 70 | Sand |  |  |
| 71 | Sand |  |  |
| 72 | Sand |  |  |
| 73 | Sand |  |  |
| 74 | Sand |  |  |

**Soil enzyme activity**

Table S2 Soil enzyme activity determination conditions

| Soil enzyme | Buffer | pH | Temperature | Time | Termination reagent |
| --- | --- | --- | --- | --- | --- |
| β-glucosidase | 50 mM acetic acid sodium acetate | 5.5 | 25 ℃ | 1 h | 0.5 M NaOH |
| Alkaline phosphatase | 50 mM borax sodium hydroxide | 9.0 | 25 ℃ | 1 h | 0.5 M NaOH |
| Urease | 50 mM phosphate phosphate | 7.0 | 25 ℃ | 2 h | Phenol sodium hypochlorite |

After passing through a 2 mm sieve, the fresh soil should be immediately stored at 4 °C and measured within 48 hours; Each sample undergoes 3 technical repetitions with a relative standard deviation of less than 5%; The standard curve is 0-100 µ M PNP (R^2^ ≥ 0.999), after deducting the blank (no soil+substrate) and the control (soil+no substrate).

**Soil nutrients analysis**

We analyzed the soil nutrients collected from the soil samples. The results showed that there were no significant differences in the SOC, TP, TK content of the surface soil between different biocrusts types in the Gurbantunggut Desert (fig S2). However, there are significant differences in the TN content in the surface soil between different biocrusts types. There are significant differences in the available nitrogen (N-NO3 and N-NH4), AP and AK contents, pH and EC in the surface soil between different biocrusts types (Fig S3, S4). They show a significant increasing trend with the development of biological crust (Fig S3, S4).


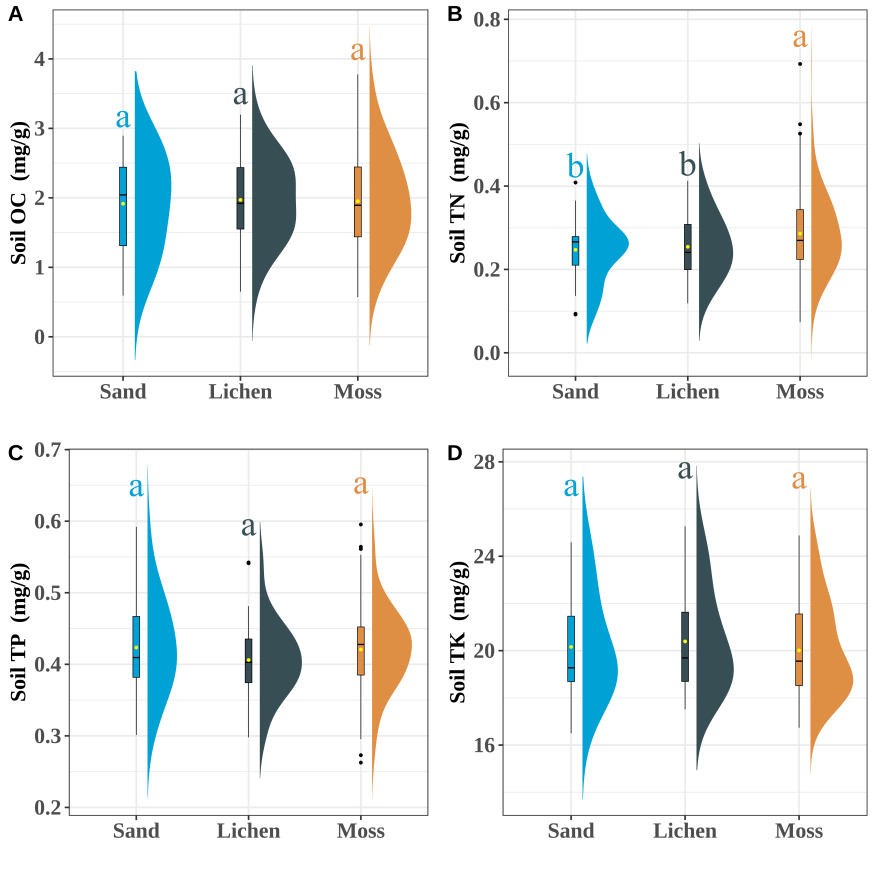


Fig S5. Changes of soil OC (A), TN (B), TP (C) and TK (D) in of different soil types (Sand: bare sand, Lichen: lichen crust, Moss: moss crust). Different letters (a and b) above the box plot indicate significant differences at *p* < 0.05.


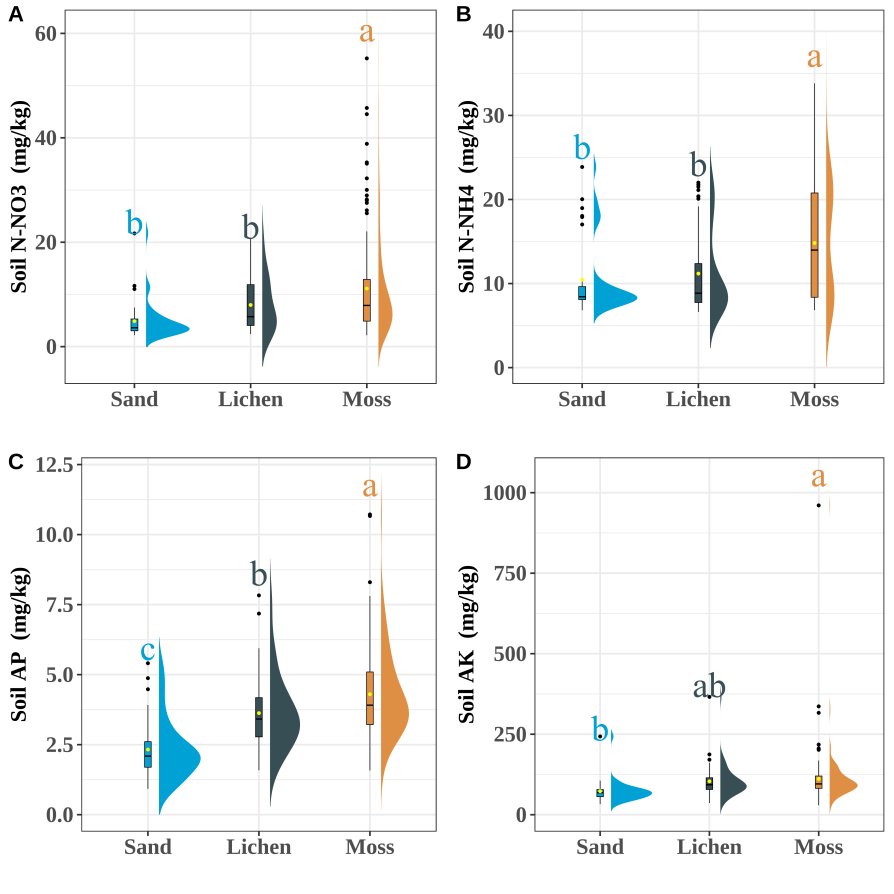


Fig S6. Changes of soil NO3-N (A), NH4-N (B), AP (C) and AK (D) in of different soil types (Sand: bare sand, Lichen: lichen crust, Moss: moss crust). Different letters (a and b) above the box plot indicate significant differences at *p* < 0.05.


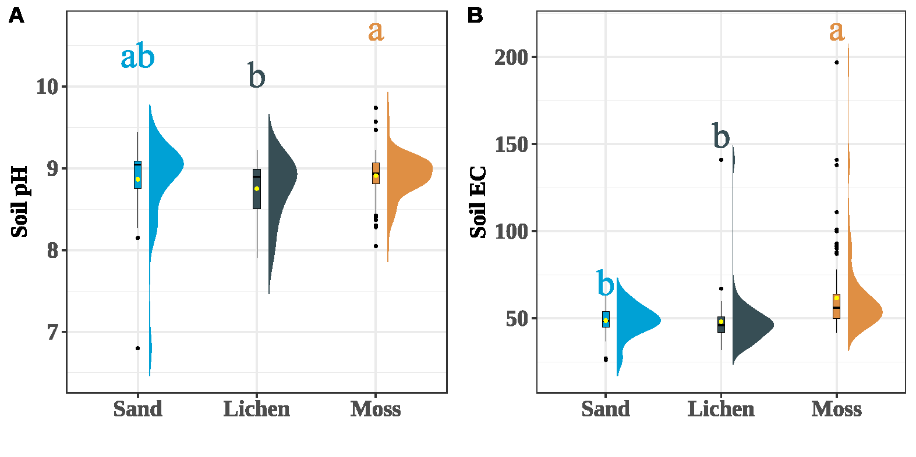


Fig. S7. Changes of soil pH (A) and EC (B) in of different soil types (Sand: bare sand, Lichen: lichen crust, Moss: moss crust). Different letters (a and b) above the box plot indicate significant differences at p < 0.05.

**Soil multifunctionality**

Normalize and perform PCA analysis on 12 original functional variables (SOC, TN, TP, TK, NO_3_⁻-N, NH_4_⁺-N, AP, AK, BG, ALP, UR, EC) to extract principal components. Retain the first 4 PCs based on eigenvalues>1, and explain 83.7% of the variance cumulatively. Calculate the weighted PC-SMF by weighting the variance contribution rate of each PC. The relationship between PC-SMF and arithmetic mean SMF is r=0.93 (p<0.001, n=74); The effect of moss vs bare sand is β=0.40 vs 0.42, and the direction and significance are completely consistent. Calculate the site level median for each functional variable. Count the number of functions above the median for each sample point. Use 'number of high functionality/total number of functions' as the Threshold SMF (range 0-1). Threshold SMF and arithmetic SMF r=0.89 (p<0.001); The peak precipitation of 163 mm and the positive and negative decomposition paths of MAT still exist. VIF<3.2 between 12 functions, maximum Pearson |r|=0.71 (SOC-TN), below the threshold of 0.8. After removing the highest correlation pair and recalculating the arithmetic SMF, the result remains unchanged with the complete data r=0.97.

**Soil quality index**

Perform PCA (KMO=0.74, Bartlett p<0.001) after standardizing the 12 original indicators. Retain the first four principal components based on eigenvalues>1, and explain 83.1% of the variance cumulatively. If Pearson | r |>0.70 for both indicators, only retain the one with the highest load (SOC and TN r=0.71, retain SOC); The final minimum dataset (MDS) contains 7 indicators: SOC, TP, AK, ALP, BG, pH, and EC. The final SQI (0-1) is obtained by weighting and summing the variance contribution rate of each indicator's PC to the cumulative variance ratio. After changing from non-linear to linear scoring, the SQI and original results have r=0.93, and the direction of the effect remains unchanged. Delete any indicator, and if the site ranking Kendall τ>0.89, it indicates that the weight and function selection are robust.

**Multicollinearity**

Calculate variance inflation factor for seven continuous variables including MAT, MAP, MAW, Rad, pH, EC, and Sand. All VIFs are less than 3.2 (maximum MAP 2.8), far below the commonly used threshold of 10, indicating that collinearity does not pose a threat. Adding a standardized MAP × MAT interaction term in the LMM framework, it was found that the slope of the MAT was slightly steep when the MAP was low, but the 95% confidence bands overlapped, which had no practical significance. Therefore, the final model retained additivity. The generalized additive mixture model was fitted using the MGCV package, and the result showed that the MAT effective degrees of freedom (EDF) were 1.02, with linear dominance. MAP edf = 2.83， It shows a significant single peak (p<0.001), consistent with the 163 mm inflection point of the segmented regression. MAW edf = 1.19， No significant bending outside of linearity.

**Evaluation of linear and non-linear**

We fitted linear and non-linear (quadratic and general additive models) regressions to the relationships between soil characteristics (SMF, SQI, SVM) and abiotic factor (MAT, MAP, MAW), and used the Akaike information criterion (AIC) to decide the model that provided the best fit in each case. This criterion penalizes model fit when more parameters (as used in non-linear regressions) are used, so that the most likely model has the lowest AIC value. In general, differences in AIC higher than 2 indicate that the models are different. Quadratic and GAM models evidence a nonlinear but continuous trend throughout the MAP gradient. We chose quadratic to synthetize the simplest case of nonlinear trend, and GAM to summarize more complex trends (through smoothing parameters).

**Thresholds detection**

We explored the presence of thresholds only when non-linear models were a better fit to the data. When fitting segmented regressions to models that are better fitted to smooth nonlinear continuous trends (such as models that best fit GAM regressions), segmented regressions evidence the point of maximum curvature of the fit. This point can be considered a threshold in the sense that it shows a peak of change in the response of the variable to MAP, even if the fit of segmented regressions is poorer than that of GAM or other nonlinear models. Discontinuous thresholds attain an overall change in the intercept, apart from the slope, and may be fitted to either step (linear regression that changes only intercept at a given point or threshold) or a combination of step + segmented regressions (stegmented; exhibits changes both in intercept and slope at a given point or threshold). We used the chngpt and gam packages in R to fit segmented/step/stegmented and GAM regressions, respectively and the segmented package to fit segmented regressions with two thresholds.
